# Supplementary material for: Neuronal Depolarization Induced RNA m5C Methylation Changes in Mouse Cortical Neurons
Source: Biology (Basel). 2022 Jun 29;11(7):988. doi: 10.3390/biology11070988 (PMC9311806; doi:10.3390/biology11070988)
Supplement: Supplementary file 1 [file biology-11-00988-s001.zip › supplementary Figures S1-S5.pptx]

## Slide 1
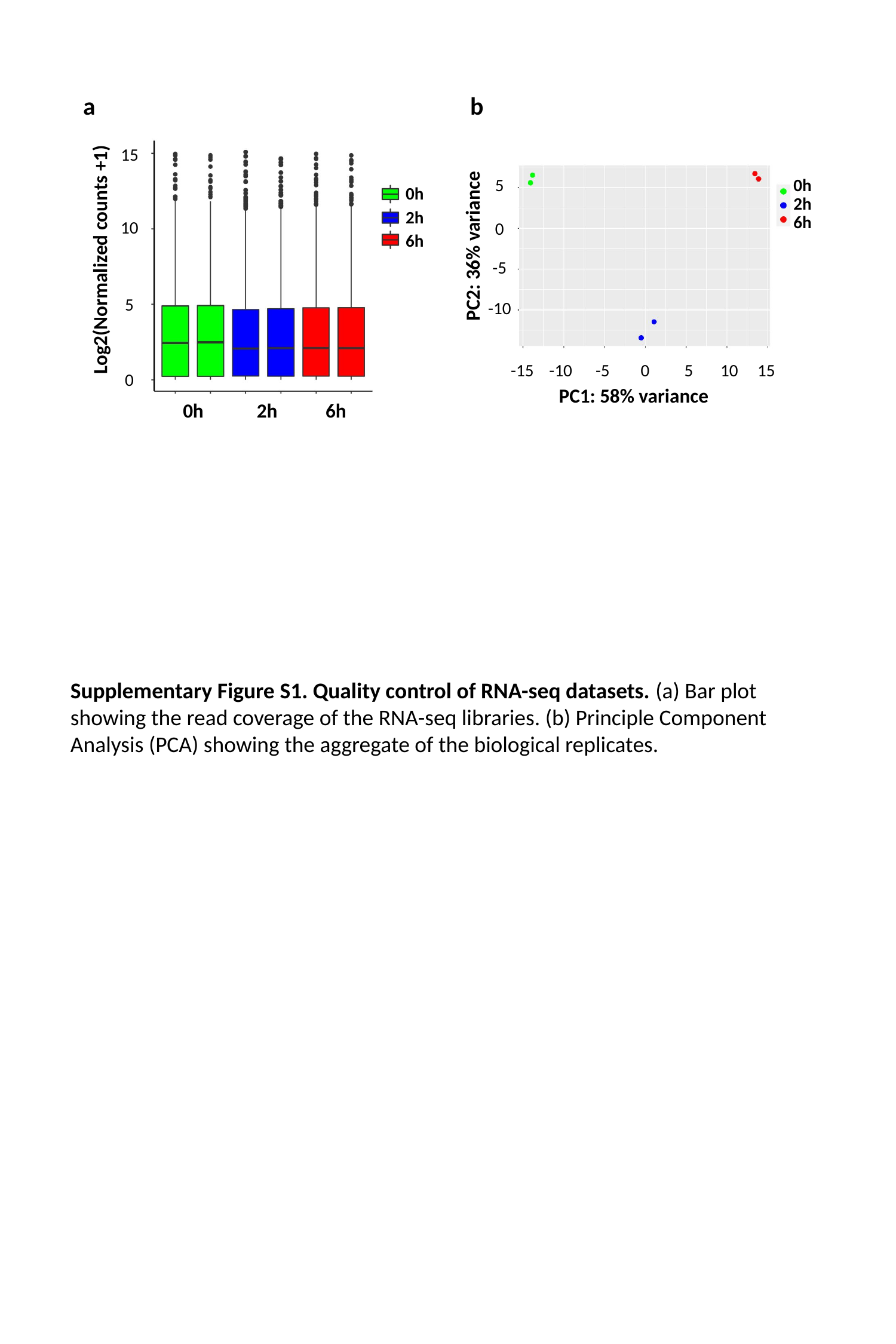

a
b
15
0h
2h
6h
10
Log2(Normalized counts +1)
5
0
0h
2h
6h
5
0h
2h
6h
0
PC2: 36% variance
-5
-10
-15 -10 -5 0 5 10 15
PC1: 58% variance
Supplementary Figure S1. Quality control of RNA-seq datasets. (a) Bar plot showing the read coverage of the RNA-seq libraries. (b) Principle Component Analysis (PCA) showing the aggregate of the biological replicates.

## Slide 2
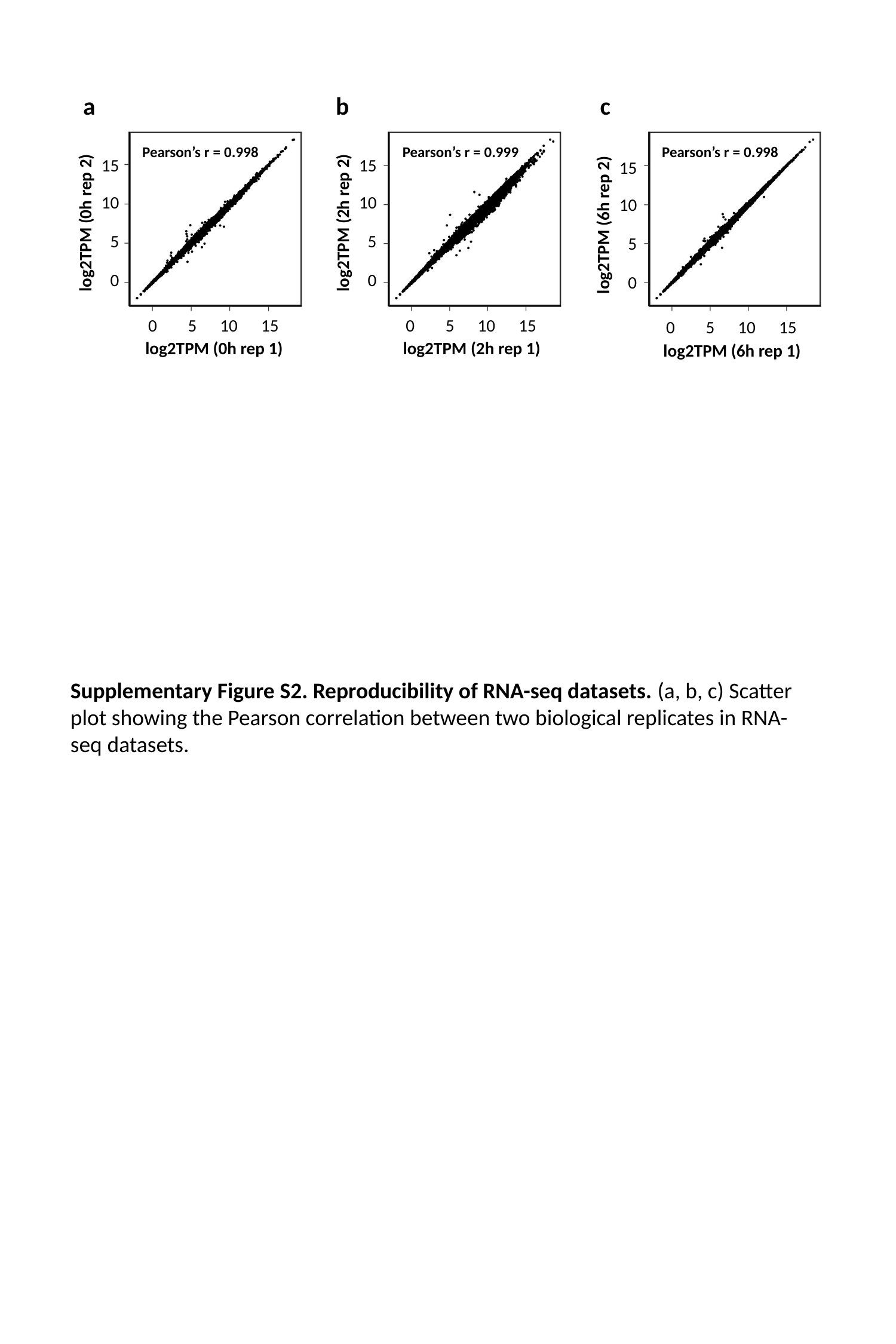

a
b
c
Pearson’s r = 0.998
Pearson’s r = 0.999
Pearson’s r = 0.998
15
10
5
0
15
10
5
0
15
10
5
0
log2TPM (0h rep 2)
log2TPM (2h rep 2)
log2TPM (6h rep 2)
0 5 10 15
0 5 10 15
0 5 10 15
log2TPM (0h rep 1)
log2TPM (2h rep 1)
log2TPM (6h rep 1)
Supplementary Figure S2. Reproducibility of RNA-seq datasets. (a, b, c) Scatter plot showing the Pearson correlation between two biological replicates in RNA-seq datasets.

## Slide 3
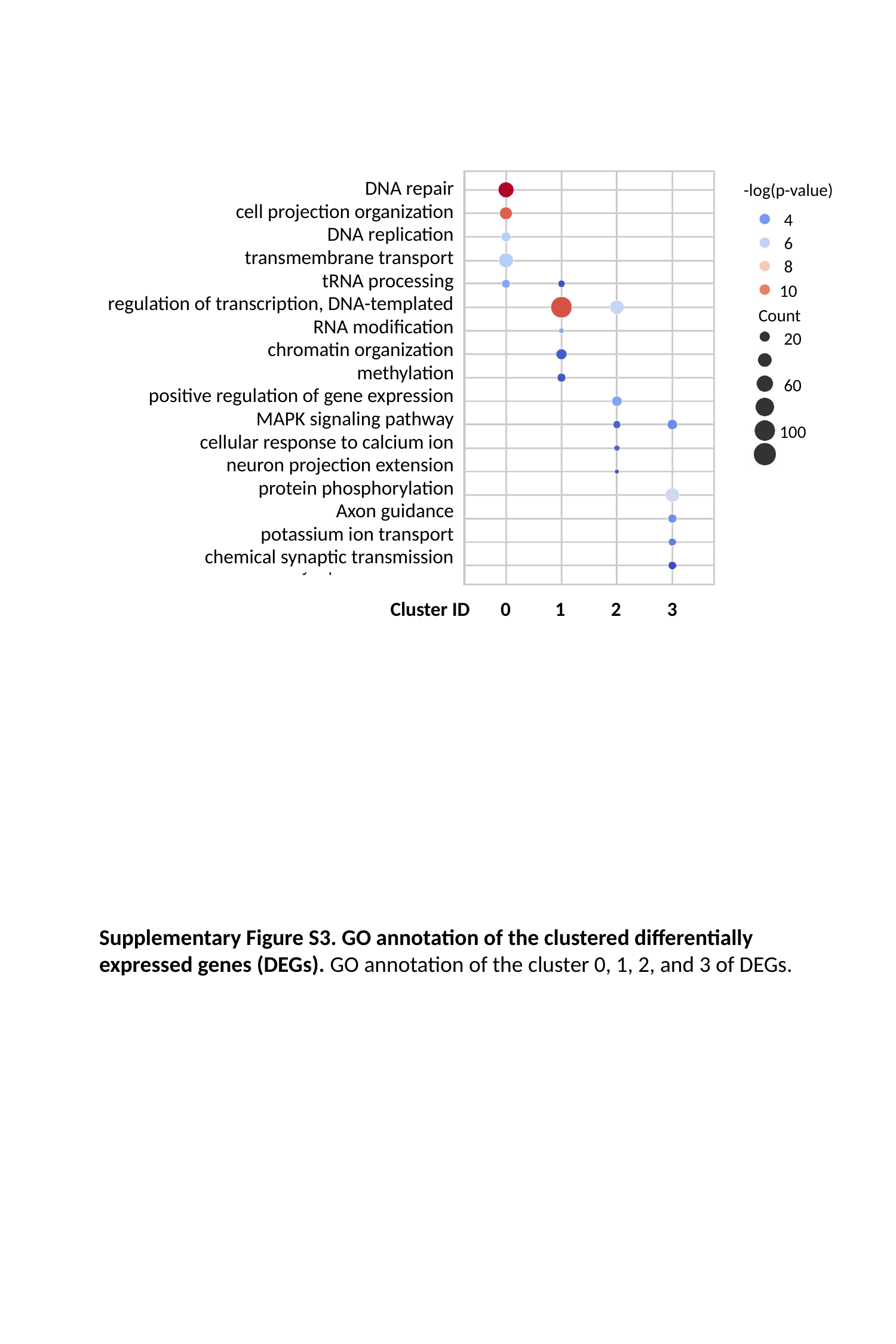

-log(p-value)
DNA repair
cell projection organization
DNA replication
transmembrane transport
tRNA processing
regulation of transcription, DNA-templated
RNA modification
chromatin organization
methylation
positive regulation of gene expression
MAPK signaling pathway
cellular response to calcium ion
neuron projection extension
protein phosphorylation
Axon guidance
potassium ion transport
chemical synaptic transmission
4
6
8
10
Count
20
60
100
Cluster ID
0
1
2
3
Supplementary Figure S3. GO annotation of the clustered differentially expressed genes (DEGs). GO annotation of the cluster 0, 1, 2, and 3 of DEGs.

## Slide 4
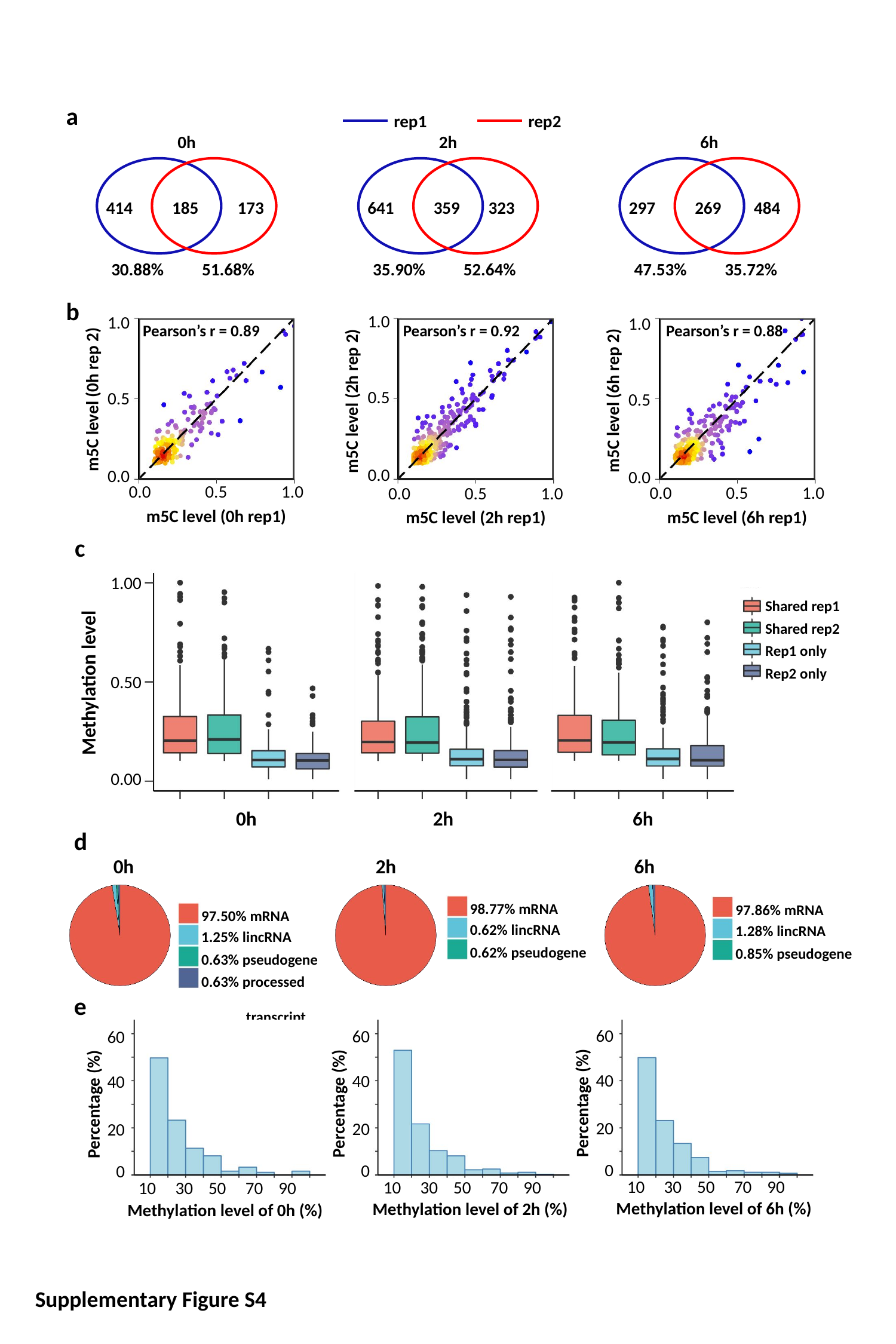

a
rep1
rep2
0h
414
173
185
51.68%
30.88%
2h
641
323
359
52.64%
35.90%
6h
297
484
269
35.72%
47.53%
b
1.0
0.5
0.0
1.0
0.5
0.0
1.0
0.5
0.0
Pearson’s r = 0.89
Pearson’s r = 0.92
Pearson’s r = 0.88
m5C level (0h rep 2)
m5C level (6h rep 2)
m5C level (2h rep 2)
0.0 0.5 1.0
0.0 0.5 1.0
0.0 0.5 1.0
m5C level (0h rep1)
m5C level (6h rep1)
m5C level (2h rep1)
c
1.00
Shared rep1
Shared rep2
Rep1 only
Rep2 only
Methylation level
0.50
0.00
0h
2h
6h
d
0h
2h
6h
98.77% mRNA
0.62% lincRNA
0.62% pseudogene
97.86% mRNA
1.28% lincRNA
0.85% pseudogene
97.50% mRNA
1.25% lincRNA
0.63% pseudogene
0.63% processed
 transcript
e
60
40
20
0
60
40
20
0
60
40
20
0
Percentage (%)
Percentage (%)
Percentage (%)
10 30 50 70 90
10 30 50 70 90
10 30 50 70 90
Methylation level of 6h (%)
Methylation level of 2h (%)
Methylation level of 0h (%)
Supplementary Figure S4

## Slide 5
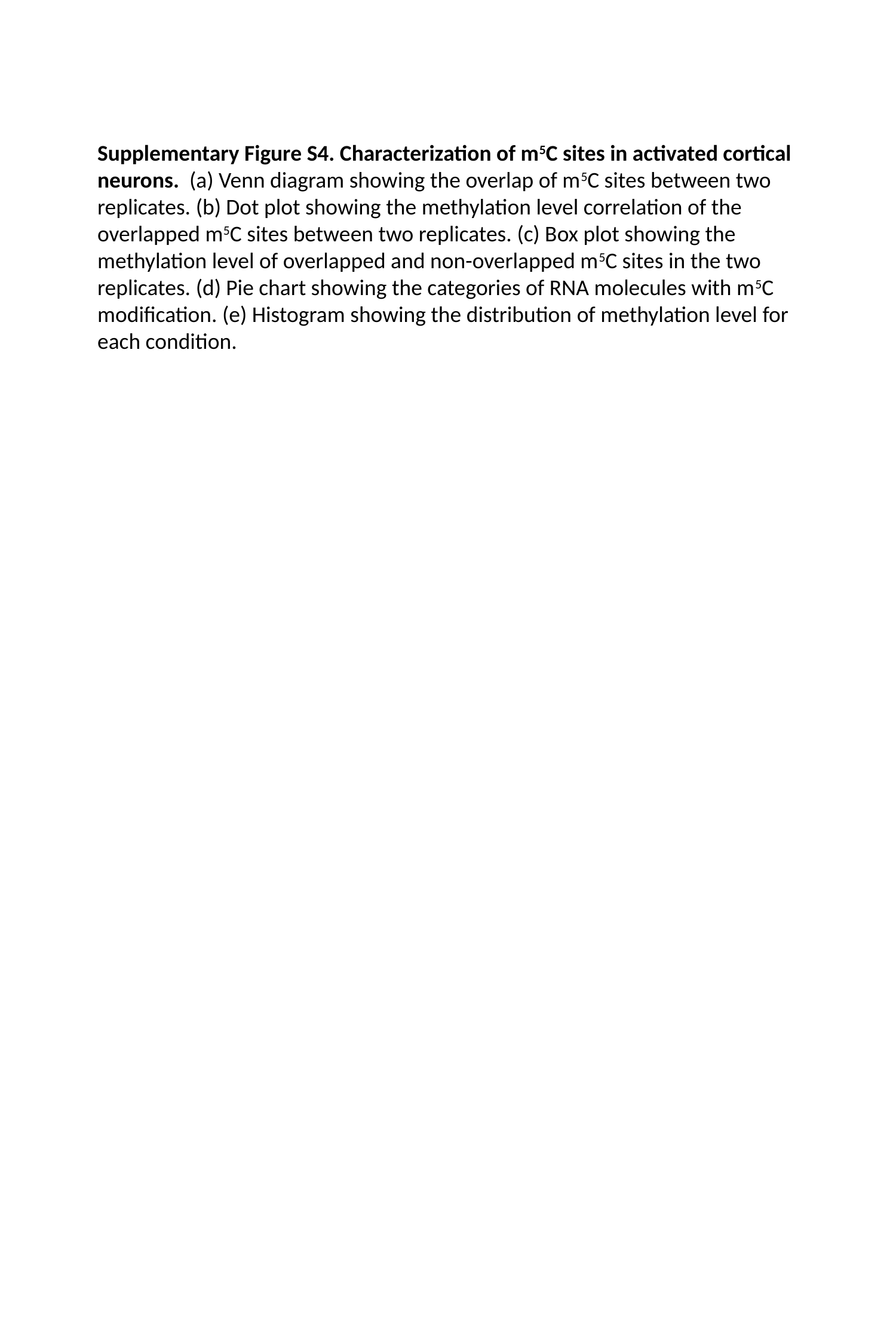

Supplementary Figure S4. Characterization of m5C sites in activated cortical neurons. (a) Venn diagram showing the overlap of m5C sites between two replicates. (b) Dot plot showing the methylation level correlation of the overlapped m5C sites between two replicates. (c) Box plot showing the methylation level of overlapped and non-overlapped m5C sites in the two replicates. (d) Pie chart showing the categories of RNA molecules with m5C modification. (e) Histogram showing the distribution of methylation level for each condition.

## Slide 6
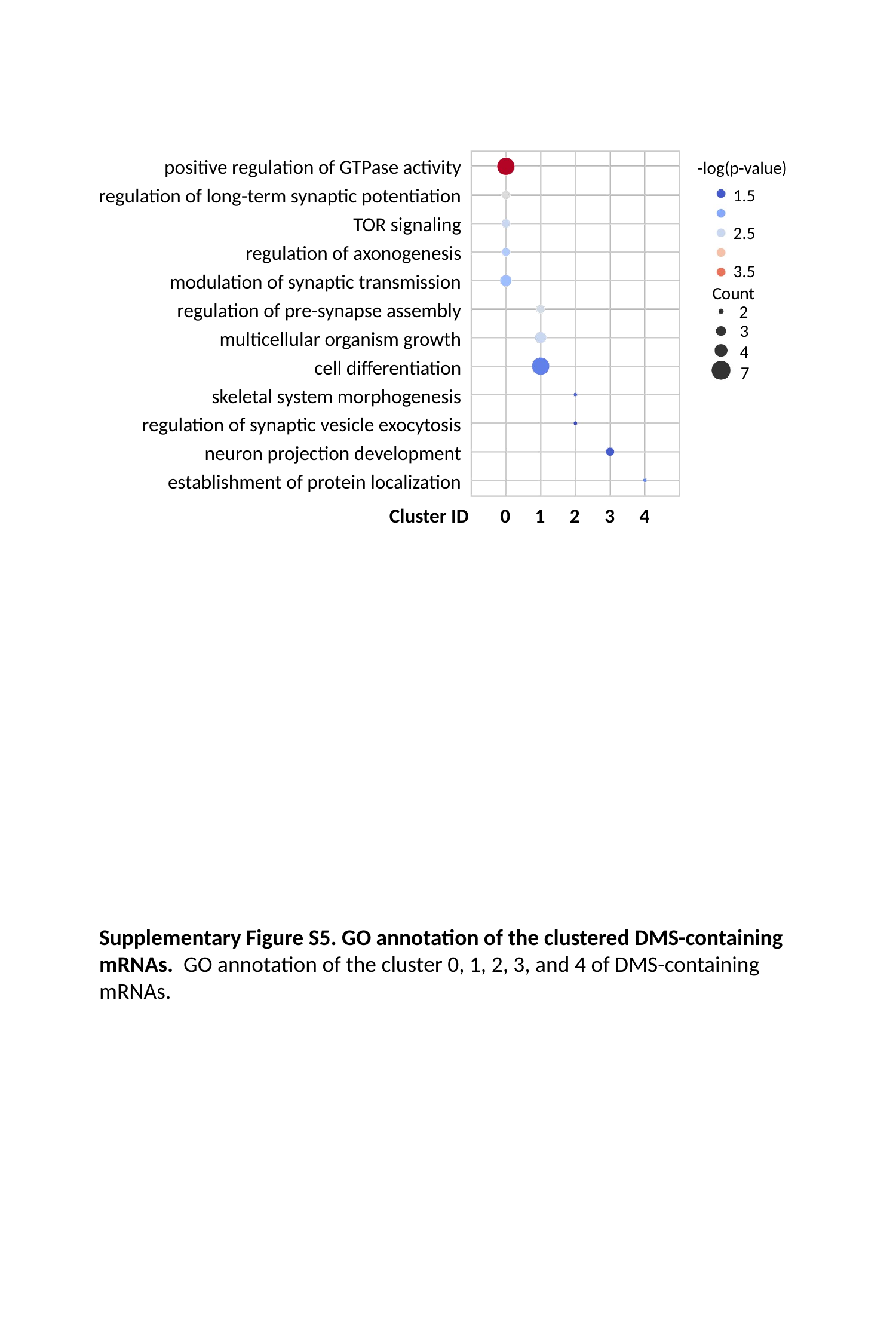

positive regulation of GTPase activity
regulation of long-term synaptic potentiation
TOR signaling
regulation of axonogenesis
modulation of synaptic transmission
regulation of pre-synapse assembly
multicellular organism growth
cell differentiation
skeletal system morphogenesis
regulation of synaptic vesicle exocytosis
neuron projection development
establishment of protein localization
-log(p-value)
1.5
2.5
3.5
Count
2
3
4
7
Cluster ID
0
1
2
3
4
Supplementary Figure S5. GO annotation of the clustered DMS-containing mRNAs. GO annotation of the cluster 0, 1, 2, 3, and 4 of DMS-containing mRNAs.
